# The Effects of Digital Addiction on Brain Function and Structure of Children and Adolescents: A Scoping Review

**DOI:** 10.3390/healthcare12010015

**Published:** 2023-12-20

**Authors:** Keya Ding, Yining Shen, Qianming Liu, Hui Li

**Affiliations:** 1 Shanghai Institute of Early Childhood Education, Shanghai Normal University, Shanghai 200233, China; keya@shnu.edu.cn (K.D.); 1000481673@smail.shnu.edu.cn (Y.S.); 1000546930@smail.shnu.edu.cn (Q.L.); 2 Faculty of Education and Human Development, The Education University of Hong Kong, 10 Lo Ping Road, Hong Kong

**Keywords:** digital addiction, adolescents, neuroimaging, brain structure, brain function

## Abstract

The escalating prevalence of studies investigating digital addiction (DA) and its detrimental impact on the human brain’s structure and functionality has been noticeable in recent years. Yet, an overwhelming majority of these reviews have been predominantly geared towards samples comprising college students or adults and have only inspected a single variant of DA, such as internet gaming disorder, internet addiction disorder, problematic smartphone use, tablet overuse, and so forth. Reviews focusing on young children and adolescents (ages 0–18), or those which amalgamate various types of DA, are decidedly scarce. Given this context, summarizing the effects of DA on brain structure and functionality during the vital developmental stage (0–18 years) is of immense significance. A scoping review, complying with the PRISMA extension for such reviews, was conducted to amalgamate findings from 28 studies spanning a decade (2013–2023) and to examine the influence of assorted forms of DA on the brains of children and adolescents (0–18 years). The synthesized evidence indicated two primary results: (1) DA exerts harmful effects on the structure and functionality of the brains of children and adolescents, and (2) the prefrontal lobe is the region most consistently reported as impacted across all research. Furthermore, this review discerned a notable void of studies investigating the neural indices of digital addiction, along with a shortage of studies focusing on young children (0–6 years old) and longitudinal evidence. This research could provide the necessary theoretical basis for the thwarting and intervention of digital addiction, a measure indispensable for ensuring healthy brain development in children and adolescents.

## 1. Introduction

Digital devices have been integrated ubiquitously into various aspects of life, including education, entertainment, and employment [1]. However, their pervasive presence has led to notable potential risks including digital overuse, and in some instances, problematic use or digital addiction (DA) [2]. Of significant concern is the susceptibility of children and adolescents, whose developmental neurobiology renders them particularly prone to problematic use of such devices, potentially leading to a range of effects spanning from inattention and cognitive deficits to declining academic performance and diminished mental and physical health [3,4,5,6]. A burgeoning body of research has elucidated the effects of digital addiction on the human brain across various populations and several reviews have addressed this topic recently [7,8]. However, most of these reviews are predominantly targeted at adult populations or college students, with a particular emphasis on a single facet of digital addiction such as internet gaming disorder or problematic smartphone use (PSU). A significant lacuna exists in the literature when it comes to reviews specifically aimed at the demographic of young children and adolescents (ages 0–18), taking into account various types of DA. Thus, fundamental questions emerge: What are the defining characteristics of children and adolescents with DA? Which brain areas are the most profoundly impacted? Are the impacts characterized more by functional or structural alterations?

Addressing these queries systematically is crucial for decoding the underlying mechanisms leading to functional and structural changes in the brains of children and adolescents with DA. These understandings will provide meaningful evidence which can underpin the development and execution of effective interventions and preventive measures. Our scoping review seeks to fill this gap by aggregating and analyzing existing research over the last decade concerning the effects of DA on brain function and structure in children and adolescent samples (aged 0–18).

This paper will commence with an overview of the concept of DA and its influence on children and adolescents. Subsequently, the objectives, methodologies and results of this scoping review will be delineated. The paper will conclude with a discussion synthesizing our findings, underscoring their implications for practical improvement and outlining potential avenues for future investigation.

### 1.1. Digital Addiction: A Global Health Issue

Digital addiction is a comprehensive term that encompasses the long-standing and emerging categories of internet addiction, gaming addiction, social media addiction, or other digital media addiction [9]. In general, “digital addiction” refers to any addictive behavior related to the use of digital devices, including cell phones, computers, the internet, video games, and social media [1]. It is worth noting that digital addiction does not necessarily involve internet use, as it includes not only addiction to online activities but also to offline activities using digital devices, such as offline gaming addiction [8,10]. In fact, with the rapid development of the internet and the accelerator of the COVID-19 pandemic, “DA” has become a worldwide health issue. Recent reports highlight the persistently high and steadily increasing global prevalence of digital users, and the age of digital addicts is showing a trend of becoming younger over time. A meta-analysis revealed that 25.89% of adolescents suffer from internet addiction [11]. The American Medical Association survey said that up to 15% of adolescents may be digital addicts, and a national study in Norway reported that the prevalence of problematic digital games was 4.1%, and the addiction rate was 0.6% [12]. Additionally, in 2013, the American Psychiatric Association included “Internet Gaming Disorder (IGD)” (both online and offline gaming) in the DSM-5 to provide diagnostic criteria and called for further research [13]. Moreover, the World Health Organization classified video game addiction as a mental disorder, either online or offline, in the 11th edition of the International Classification of Diseases in 2018 [14]. Therefore, digital addiction has become a serious global health issue.

### 1.2. The Effects of Digital Addiction on Children and Adolescents

Previous studies have revealed the negative effects of digital addiction on children and adolescents in various dimensions, including physical health, emotional well-being, cognitive performance, brain function, and brain structure. Aziz et al., (2021) have highlighted that internet gaming disorder (IGD) and internet addiction (IA) could lead to impairments in vision and hearing among adolescents, as well as an increased risk of obesity and muscle pain [15]. Studies on the socio-emotional development of preschoolers have shown that DA negatively impacts children’s social skills and social relationships, showing more aggressive behavior and a high frequency of depression and anxiety [16,17,18]. Farchakh et al., (2020) noted that video game addiction was significantly negatively correlated with children’s attention, memory, and problem-solving skills [19]. Moreover, DA has been proven to influence the human brain functionally and structurally. An EEG study showed the reward processing system and cognitive control ability of “digitally addicted” adolescents were altered [20]. Li et al., (2014) assessed the response inhibitory network by fMRI and found that adolescents with internet addiction have impaired function connectivity in the basal ganglia of the frontal lobe [21]. Studies focusing on structural brain impairment have pointed out that alterations in brain structure (gray/white matter volume and cortical thickness) due to digital addiction [7,22,23]. In particular, Yoo et al., (2021) found that adolescents with problematic smartphone use exhibited reduced caudate nucleus volume compared to the control group [24]. Taken together, these previous studies have revealed the negative impact of digital addiction on children and adolescents from multiple aspects (e.g., physical, emotional, cognitive, and brain structure and function). Nevertheless, there have been no comprehensive assessments undertaken to integrate these pieces of evidence and furnish a substantiated conclusion, thereby necessitating the execution of this scoping review.

### 1.3. Research Objectives

Over the previous ten years, technological and digital advancements have not only ushered in the integration of digital devices within the educational sector but have also facilitated the emergence of digital addiction. This evolving malady is becoming increasingly prevalent, impacting both juveniles and adults. Comprehensive reviews have already investigated the repercussions of digital addiction on adults, with an increased focus on tertiary students [25,26]. However, there is a noticeable lack of exploration into its impact on cognitive function and structure, particularly on infants, children, and adolescents (0–18 years)—a demographic identified as highly susceptible to digital addiction. Furthermore, studies concerning the effect of digital addiction on distinct cerebral regions remain fragmented and deficient in systematic consolidation. To address this void in the literature, the current study aims to synthesize and critically analyze investigations centered on the functional and structural brain implications of digital addiction within children and adolescents. This will be accomplished by sorting various neuroimaging techniques, summarizing the research findings, and offering a theoretical foundation for counteractive measures during the brain’s highly impressionable and malleable stages. This scoping review will not only systematically summarize the latest findings of the negative impacts of DA on children’s brain function and brain structure but also provide the implications for parents and educators to intervene appropriately and timely in children and adolescents with DA. Accordingly, the study is led by the following research questions:

1. Does digital addiction cause functional and structural changes in the brains of children and adolescents (ages 0–18)?
2. Which specific regions of the brain are most significantly affected by digital addiction in children and adolescents?

## 2. Methods

The present study aimed to investigate the breadth and depth of existing research on the impact of DA on children’s brains. With the rapid development of brain imaging techniques, the number of studies in this field is surging. Therefore, a scoping review was conducted to focus on the latest results during the past decade. The present scoping review followed Arksey and O’Malley’s scoping review framework, which included five steps: identifying the research questions, identifying relevant studies, studies selection, charting data, collating, summarizing, and reporting [27]. Through the five steps, this study aimed to review studies relevant to the impact of DA on children’s brains published from 2013 to July 2023, classify research results by different categories (neuroimaging technique, functional or structural impact…), and identify the brain region affected most.

### 2.1. Identifying Relevant Studies

The electronic search was undertaken on online databases, including Google Scholar, Web of Science, Science Direct, and PubMed. The last search was conducted on 11 August 2023. When identifying relevant studies, the following search terms with two Boolean operators (“AND” and “OR”) were used: (“digital” OR “internet” OR “internet game” OR “smartphone” OR “screen” OR “tablet”) AND (“addiction” OR “disorder” OR “excessive” OR “heavy” OR “pathology”) AND (“brain function” OR “brain structure” OR “neuroimaging” OR “gray matter” OR “white matter” OR “frontal” OR “temporal” OR “parietal” OR “occipital” OR “fNIRS” OR “MRI” OR “EEG”) AND (“adolescent” OR “teenager” OR “young children” OR “children” OR “youth”).

### 2.2. Studies Selection

To ensure that only full-text, peer-reviewed journal articles were included, the present study set inclusion/exclusion criteria.

The criteria for inclusion were as follows: (1) published from January 2013 to July 2023; (2) peer-reviewed scholarly journal articles; (3) the written language of the article was English; (4) the age of all participants were from 0 to 18 years old; experiment group participants should be diagnosed with at least one type of digital addiction (internet addiction, internet gaming addiction, cyber addiction, excessive internet use…) or self-reported to have suffered from typical components of at least one type of digital addiction; (5) using brain imaging technique (MRI, fNIRS, EEG, PET); and (6) the research topic was about how DA influenced children’s brain function and brain structure.

The articles were excluded if they met the following: (1) reviews, theoretical studies, editorials, or secondary data analysis; (2) not available in English; (3) participants aged over 18 years old or with special needs; children whose digital addiction or typical components of digital addiction were not mentioned; (4) articles that do not focus on children’s brain or only presented behavior results.

**Figure 1:**
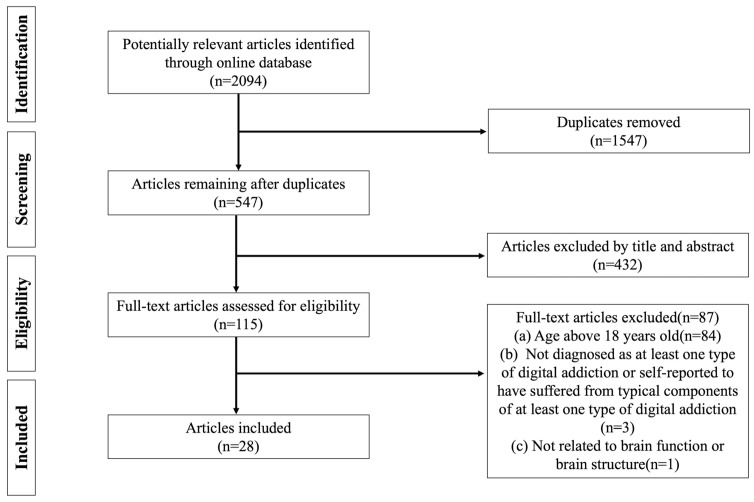
PRISMA flow diagram of literature search and data charting.

Based on the inclusion/exclusion criteria, 2094 potentially relevant articles were found during the initial search. After removing 1547 duplicate articles by Endnote, a total of 547 articles remained and were imported into Covidence. After that, the title and abstract were screened and 430 irrelevant articles were excluded. Next, 87 of 117 articles were removed for the following reasons: (a) age above 18 years old (n = 84); (b) not diagnosed as at least one type of digital addiction or self-reported to have suffered from typical components of at least one type of digital addiction (n = 3); (c) not related to brain function or brain structure (n = 1). Finally, 28 qualified articles were included in the present scoping review. To avoid including debatable studies, three screeners all checked the coding and agreed on the final 28 articles included in this review. Importantly, the present study was conducted following the Preferred Reporting Items for Systematic Reviews and Meta-Analyses (PRISMA) guideline (Figure 1).

### 2.3. Charting Data

Table 1 (and those in the Appendix A and Appendix B) presents the characteristics of 28 selected studies, including author(s), year of publication, country, neuroimaging technique, sample size, participants’ details (age, gender, diagnosis criteria), research design (addiction type, research paradigm, behavioral assessment), significant results relevant to the impact of DA on brain of children and adolescents, and limitations.

### 2.4. Collating, Summarizing and Reporting

This scoping review categorized 28 concluded studies by type of neuroimaging technique (MRI, EEG, fNIRS…) and focused on four themes: demographic information of the studies, the impact of DA on brain function of children and adolescents, impact of DA on brain structure of children and adolescents, and the brain region affected most. Please refer to Table 1 and Appendix A and Appendix B for details of these studies.

## 3. Results

### 3.1. Demographic Information

#### 3.1.1. Year of Publication

All 28 studies included were published during the decade (2013.1–2023.7). Furthermore, 14 out of 28 (50%) were published within the past 5 years, with 4 in 2018, 4 in 2020, 5 in 2021, and 1 in 2023. The others (50%) were relatively more distant: 5 in 2013, 4 in 2014, 3 in 2015, and 2 in 2017. Among all years of publication, 2013 and 2021 witnessed the most publication.

#### 3.1.2. Country

A majority of studies (27 out of 28) were conducted in Asia, with 14 from Republic of Korea, 12 from China, and 1 from Japan. The other study was from America.

#### 3.1.3. Participants

A total of 1821 participants were recruited in 28 studies, and the sample size of a single study ranged from 22 to 507. Although the age of the participants was from 4 to 18, most studies (26 out of 28) focused on children aged 12 to 18. Among 19 studies that reported the gender of participants, more male participants were included than female (685 males: 240 females). Among the 28 studies, 27 of them used a cross-sectional method and 1 collected both cross-sectional data and longitudinal data.

#### 3.1.4. Type of Digital Addiction

We found 5 types of digital addiction were included in the 28 studies: internet gaming addiction (16, 57.1%), internet addiction (6, 21.4%), smartphone addiction (4, 14.3%), heavy use of tablets (1, 3.6%), and high media multitasking (1, 3.6%) (Table 2).

#### 3.1.5. Research Paradigm

In the present review, 8 out of 28 studies were task-based studies. As an effective way to estimate inhibitory control, the go/no-go task was used most (3 studies). Three studies designed a button reaction task and one study used The Dimensional Change Card Sort (DCCS). The rest combined the N-back task, color Stroop task, and number–letter task to test three domains of executive function (working memory, inhibitory control, and cognitive flexibility), respectively.

#### 3.1.6. Neuroimaging Technique

Table 3 presents the neuroimaging techniques used by 28 studies. In particular, 9 studies used functional Magnetic Resonance Imaging (fMRI), 10 studies reported resting-state functional Magnetic Resonance Imaging (rsfMRI), 6 studies adopted Magnetic Resonance Imaging (MRI), 2 studies employed functional Near-Infrared Spectroscopy (fNIRS), and 1 study reported Electroencephalogram (EEG).

### 3.2. Neuroimaging Evidence

#### 3.2.1. EEG/ERP Evidence

The present study only includes one study that recruited the EEG to assess the brain electric activity of IGD adolescents. Study 19 [20] reported the dysfunction of the inhibitory–control system and reward–approach system of IGD adolescents using the go/no-go task (to assess inhibitory control) and gambling task (to assess reward processing). IGD adolescents had a higher degree of sensation seeking and novelty seeking and were more likely to lead to negative outcomes. High IGD symptoms were related to low accuracy and reduced P3 amplitude in the no-go task, which indicated the impaired inhibitory-control system of the IGD group. Furthermore, although both the control group and the IGD group were more inclined to avoid risk after positive outcomes than after negative outcomes, the IGD group demonstrated lower feedback-related negativity magnitude (a neural indicator of reduced reward sensitivity) after positive outcomes than the control group. The study also found that the relationship between the inhibitory–control system and reward–approach system was disrupted in the IGD group.

#### 3.2.2. MRI Evidence

Six MRI-based studies that tested the volume and integrity of certain brain areas were included in this review. First, Study 1 [28] and Study 28 [24] both recruited problematic smartphone users (PSU/SP) and measured the altered volume of certain brain areas. Study 1 [28] screened 87 participants and categorized them into the PSU group and the control group using the Smartphone Addiction Proneness Scale (SAPS). As a result, a significantly smaller volume of the superior cerebellar peduncle (SCP) was found in the PSU group. The volume of the SCP was negatively correlated with the SAPS score. Using the same diagnosis criteria, this research team tested the caudate volume of PSU in Study 28 [24], finding that the decrease in caudate volume on both sides reached a significant level. In addition, left caudate volume was negatively correlated with impulsivity traits and SAPS scores.

Second, Study 10 [22], Study 21 [23], Study 23 [29], and Study 27 [5] focused on children with internet gaming disorder/internet addiction (IGD/IA). Study 10 [22] recruited 30 male adolescents with IA using the Young Internet Addiction Scale (YIAS). As a result, reduced thickness in the right lateral OFC and pars orbitalis was found in the group with IA, which indicated impaired cognitive flexibility and inhibitory control. Study 21 [23], which categorized participants by the internet addiction test (IAT), found that IGD tendency was negatively correlated with the gray matter volumes (GMVs) of the bilateral postcentral gyri (postCG), the left precentral gyri (preCG), the left posterior midcingulate cortex (pMCC), and the right middle frontal gyrus (MFG). The high IAT score subgroup (score > 50) had lower GMVs of the bilateral postCG, the left preCG, the left pMCC, and the right MFG than the low IAT score subgroup (score ≤ 50). Study 23 [29] also measured the regional gray/white matter volume (rGMV/rWMV) of young excessive internet users, reporting a decreased rGMV in certain regions that related to attention, executive function, the reward processing system, and the language system (orbitofrontal and lateral prefrontal areas, the insula, the putamen, the pallidum, the thalamus, medial temporal areas, the anterior temporal pole, and the cerebellum). Study 27 [5] collected VBM and TBSS data and reported that IGD adolescents exhibited gray and white matter integrity which was impaired in some areas of the prefrontal lobe (the OFC, the SMA).

#### 3.2.3. fMRI Evidence

Using fMRI, nine studies revealed the negative impact of DA on adolescents’ brains. In particular, seven studies reported fMRI evidence of internet gaming disorder in children (IGD/IGA). Study 3 [30] designed a button reaction to test the brain activation and emotional processing of adolescents with IGD. During the swear word condition, a reduced activation in the right orbitofrontal cortex (OFC) and the dorsal anterior cingulate cortex (dACC) related to social rejection were found. These two areas were related to cognitive control and social rejection, respectively. Study 2 [31] reported significant activation in the medial prefrontal (mPFC) and anterior cingulate (ACC) of the group with IGD when thinking about their game characters compared to when thinking about themselves. The ACC activation was correlated with symptom severity. Study 12 [32] revealed a similar result to Study 2, finding that the FC from the left cingulate to both lentiform nuclei was decreased and negatively correlated with YIAS scores.

Hong and his team conducted research about the impaired functional connectivity in male adolescents with IGD. In particular, Study 9 [33] reported that IGD was related to reduced functional connectivity in cortico–striatal circuits and impaired connection in cortico–subcortical circuits (24% with prefrontal and 27% with parietal cortex). Another study [34] that this research team conducted found a reduced dorsal putamen functional connectivity with the posterior insula-parietal operculum in adolescents with IGD. Moreover, functional connectivity between the dorsal putamen and bilateral primary somatosensory cortices was reported to be correlated with online game time.

Based on the go/no-Go research design, Study 6 [35] compared the neural response of normal adolescents and adolescents with IGA and reported abnormal hyperactive neural responses in several areas of groups with IGA during no-go trials. Brain areas mentioned in the result were mostly located in the prefrontal, including the left superior medial frontal gyrus, right superior/middle frontal gyrus, right anterior cingulate cortex, left precentral gyrus, left precuneus, and cuneus. Such a result suggests that the participants with IGA put more effort into the response–inhibition system than the control group. Abnormal hyperactive function of the left superior medial frontal gyrus was positively associated with BIS and CIAS total score across IGA participants.

Study 7 [36] examined the cerebral blood flow (CBF) of adolescents with IGA. This study explored a higher CBF in the left inferior temporal lobe, left para hippocampal amygdala, right medial frontal lobe/anterior cingulate cortex, bilateral insula, right middle temporal gyrus, right precentral gyrus, left supplementary motor area, left cingulate gyrus, and right inferior parietal lobe as well as lower CBF in the left middle temporal gyrus, left middle occipital gyrus, and right cingulate gyrus in IGA groups.

Using a task-based research paradigm, Study 13 [37] and Study 17 [21] measured the activation of certain brain regions of adolescents with IAD. Study 13 (Kim et al., 2014) designed a right-left discrimination task in which each task belonged to performance feedback (PF), social reward (SR) (such as compliments), monetary reward (MR), or no reward (NR), respectively. Almost no activation was found in the subcortical system of those with AIA. However, for the PF–NR contrast, a significant activation in the DLPFC was found in the IAD group. Moreover, a negative correlation was found between the level of activation in the left superior temporal gyrus (BA 22) and the duration of internet use. Using the go–stop task, Study 17 [21] discovered a weaker activation of the inferior frontal gyrus (IFG) and the striatum, located in the response inhibition network of corn brain regions. Such results indicate the impaired response–inhibition system in adolescents with IAD.

#### 3.2.4. rsfMRI Evidence

Ten studies provided rsfMRI evidence to demonstrate the negative impact of DA on functional connectivity (FC). Firstly, Study 4 [38] and Study 22 [39] both provided rsfMRI evidence of alteration in the FC of problematic smarter users (PSU/SP). Study 22 [39] reported rsfMRI evidence of altered resting-state functional connectivity (rsFC) of adolescents with PSU. Specifically, the PSU group showed a reduced rsFC between the right inferior frontal gyrus and limbic areas (the bilateral parahippocampal gyrus, the left amygdala, and the right hippocampus), which was negatively associated with the severity of problematical smartphone use symptoms and the degree of self-control.

Moreover, a similar alteration in FC between certain areas of the prefrontal was reported in Study 4 [38]. Compared with HC, lower functional connectivity between the right orbitofrontal cortex (OFC) and nucleus accumbent (NAcc), and between the left OFC and midcingulate cortex (MCC) were found in the SP. Greater FC between the MCC and NAcc was found in SP. Furthermore, Study 5 [40], Study 8 [6], Study 15 [41], and Study 16 [42] revealed the rsfMRI evidence of altered brain structure and brain function of internet gaming disorder children (IGD/IGA). Study 5 [40] tested the FC of adolescents with IGA, finding increased FC in the bilateral cerebellum posterior lobe and middle temporal gyrus, and reduced FC in the bilateral inferior parietal lobule and right inferior temporal gyrus in adolescents with IGA. Additionally, connectivity with the PCC was positively correlated with CIAS scores in the right precuneus, posterior cingulate gyrus, thalamus, caudate, nucleus accumbent, supplementary motor area, and lingual gyrus. It was negatively correlated with the right cerebellum anterior lobe and left superior parietal lobule. Study 8 [6] examined the rsFC between the left medial OFC and the putamen and found it significantly lower in the IGD group. The FC values of the IGD group were negatively associated with the BIS-11 scores. Furthermore, functional alternations were observed in several prefrontal areas (including the right medial OFC, the bilateral SMA, and the left ACC) and basal ganglia regions (the bilateral putamen) in addictive disorders, including IGD.

Additionally, Study 16 [42] tested the FC of the salience network and default mode network (DMN) with the left posterior superior temporal sulcus (pSTS) and found a significant difference between adolescents with IGD and normal adolescents. Such aberrance was correlated to the tendency of addiction and self-reported cognitive problems. Collecting neuroimaging data twice (at the baseline and after one year), Study 15 [41] found that compared with pro-gamers, adolescents with IGD showed hyperactivity within the left orbitofrontal cortex.

Last but not least, in relation to internet addiction disorder (IAD/IA), Study 14 [43], Study 24 [44], Study 25 [45], and Study 26 [46] all tested the FC of adolescents with IAD. Study 14 [43] and Study 24 [44] reported both the inter-hemispheric connections and intra-hemispheric connections. In particular, Study 14 [43] found increased positive rsFC between the left insular−right middle temporal gyrus, the right hippocampus−right precentral gyrus, the right amygdala−right precentral gyrus, and the right parietal operculum cortex. Study 24 [44] reported a reduced inter-hemispheric FC in the right frontoparietal network (FPN), whereas increased intra-hemispheric FC of the left FPN was found in adolescents with IA. Reduced FC was also found between the salience network (SN) and DMN. Study 26 [46] revealed the impaired inter-hemispheric connections of the IAD group, finding the disrupted FC between regions located in the frontal (right middle OFC), occipital (the left fusiform gyrus), and parietal (the left angular gyrus) lobes. This study also assessed the correlation between abnormal FC and the IAD severity. Study 25 [45] found reduced rsFC in the bilateral prefrontal lobe of IGD adolescents, providing neuroimaging evidence of cognitive impairment in adolescents with IGD. 

#### 3.2.5. fNIRS Evidence

Reporting the impact of high media multitasking and heavy tablet use, respectively, two fNIRS studies (Study 18 [4] and 20 [47]) were included in this review. In particular, Study 20 [47] found that two dimensions (working memory, inhibitory control) of the executive function of the high media multitaskers (HMMs) were more impaired than the low media multitaskers (LMMs) through the self-reported measurements. Moreover, prefrontal activation of the HMMs was greater than the LMMs during the 2-back and color Stroop task. Study 18 [4] categorized the participants into three groups: heavy-user, low-user (removed), and non-user by Home Learning Environment and Practice Survey (HLEP). Using the DCCS task, this study reported a significantly higher accuracy of non-users than heavy users in the DCCS task. Before the 7th second, a significant increase in HbO and decrease in HbR were found in the BA9 of non-users. From the 12th second to the 20th second, a significant decrease was found in the BA9 in heavy users during the DCCS task, which suggested the negative influence of tablet use on young children’s brain structure and brain function.

In conclusion, a comprehensive review of nineteen studies reveals pivotal insights into the detrimental effects of digital addiction (DA) on children’s brains. Notably, seven studies demonstrated abnormal brain activation, while twelve studies established impaired functional connectivity as a significant concern. Additionally, evidence indicates that DA can influence the brain’s physical structure. In particular, five articles reported decreased volume, while six articles highlighted reduced cortical thickness. However, only a solitary study delved into the consequences of DA in altering cerebral blood flow. Further research is suggested to consolidate these preliminary findings.

## 4. Discussion

This scoping review offers a comprehensive synthesis of recent findings on the influence of various digital addiction (DA) forms on brain structure and functionality in children and adolescents, presenting a contemporary overview of the relationship between DA and neural development in this population. This discussion will delve into the amalgamated insights from the 28 evaluated studies. First, it will contemplate the detrimental effects of DA on a child’s brain in two aspects: functional and structural impacts. Second, it will meticulously identify and discuss the prefrontal lobe as being the most frequently reported region among affected brain areas. Last, it will draw some meaningful conclusions, acknowledge the limitations, reflect on the implications for future research, and provide a roadmap for subsequent studies.

### 4.1. Evidenced Impact on Brain Function

#### 4.1.1. Abnormal Brain Activation

Seven studies in the present review provide evidence of abnormal brain activation in adolescents with DA, showing abnormal alteration patterns in different brain regions. First, weaker activation of the inferior frontal gyrus (IFG) and striatum of the control inhibitory network was found during an inhibitory control task, suggesting that the response inhibition system is impaired in adolescents with IAD [21,48]. Second, many studies have found higher neural activation in prefrontal regions in digital addicts compared to typical developmental samples. For example, in go/no-go reward experiments, the neural responses of adolescents with IGD are over-activated in prefrontal brain areas including the OFC, DLPFC, the left superior medial frontal gyrus, right superior/medial frontal gyrus, right ACC, left precentral gyrus, left precuneus gyrus, left inferior temporal lobe, left parahippocampal gyrus/amygdala, right medial frontal cortex, right PCC, and bilateral insula. These results suggest that adolescents with IGD invested more effort in response–inhibition systems [35,37,41]. These findings are in line with several previous studies. For example, Dong, Huang, and Du recruited a guessing task to assess the brain function of adults with IGD and found that compared to non-IGD adults, adults with IGD revealed a higher activation in the OFC [49]. Moreover, abnormal brain activation is also found in other addictions like cocaine, alcohol, and drugs, with hyperactivation found in the DLPFC and IFG [50,51]. Most of these studies involved psychological experimental paradigms to assess brain activation during cognitive activities, such as the go/no-go task, the N-back task, the color Stroop task, the number–letter task, and the DCCS [4,35,47]. In contrast, those studies that employed rsfMRI and were not based on tasks directly observed the functional connectivity of certain brain regions such as the prefrontal lobe [6,40].

#### 4.1.2. Impaired Functional Connectivity

A dozen research articles have utilized fMRI to assess functional connectivity (FC) in children with digital addiction. Our synthesized review concludes that adolescents exhibiting internet gaming disorder (IGD) show compromised FC within the cortico–subcortical circuits. Particularly, a distinct downturn in FC between the dorsal thalamus and the bilateral primary somatosensory cortex in adolescents portraying IGD has been identified to be directly proportional to online gaming duration. This insight echoes the findings reported in a recent review [52]. The magnetism of digital gaming can be partially attributed to its capacity to offer stimulating, immersive, and thrilling experiences, especially for the adolescent demographic. Succumbing to such allure leads to changes in FC across specific brain regions responsible for advanced cognitive functions. Consequently, these individuals require enhanced brain FC for tasks necessitating elevated attention, inhibition, and planning [53].

However, consensus is still lacking in research on whether FC is unequivocally reduced in adolescents with digital addiction. A few studies have reported an augmentation in FC in regions like the bilateral cerebellum lobe and the middle temporal gyrus in adolescents with DA. The cerebellum plays a crucial part in craving induction triggered by IGD, and participation in planning, execution, and memory processes [54]. Similarly, the middle temporal gyrus significantly contributes to language comprehension and semantic memory processing [55,56]. The precise mechanisms leading to this amplified FC remain elusive, but a validated correlation exists between enhanced FC in the right cerebellum anterior lobe and lower Chen Internet Addiction Scale (CIAS) scores [40]. In conclusion, findings from these fMRI-centric research efforts indicate that excessive use of digital devices precipitates abnormal brain FC in children and adolescents.

### 4.2. Evidenced Impact on Brain Structure

#### 4.2.1. Decreased Volume

Changes in brain structure have been documented in children and adolescents struggling with digital addiction. Specifically, five studies have reported a reduction in gray/white matter volume (GMV/WMV) in adolescents with this issue. Highlighting an early study, the detrimental effects of problematic smartphone use (PSU) were identified through reduced volume in the caudate nucleus and cerebellum, both of which engage in executive function, reward processing, and sensorimotor activities [57]. These findings suggest that individuals with PSU grapple to employ cognitive capabilities, such as inhibition, leading to dysfunctional smartphone utilization.

Similar volume diminutions to those associated with PSU are also identified in other forms of digital addiction, including internet gaming disorder (IGD) and internet addiction (IA), particularly affecting young adolescents. For instance, three studies unveiled decreases in GMV/WMV in several regions of the cerebral cortex, namely the orbitofrontal cortex (OFC), supplementary motor area (SMA), pre/postcentral gyrus (pre/postCG), posterior midcingulate cortex (PMCC), dorsolateral prefrontal cortex (DLPFC), and basal ganglia circuitry [5,23,29]. The OFC, a principal area for decision making, emotion processing, and inhibition [58,59] may lead to decreased behavioral control, triggering higher urges to play internet games upon a reduction in volume. In particular, the SMA, positioned within the motor cortex of the frontal lobe, is engaged in regulating movements [60]. As reported by Weng et al., (2013), reduction in this region is ascribed to repetitive motor actions, such as clicking [5]. This is supported in a meta-analysis from Qin et al., (2020), which identified compromised SMA integrity in behavioral addictions like pathological gambling [61]. The pre/postcentral gyrus (preCG/postCG), responsible for motor planning and sensory information integration, exhibited impairments in adolescents with IGD [62,63]. While direct correlations between digital addiction and GMV in pre/postCG are scant, existing evidence parallels this impairment with substance abuse’s effects on the GMV in these areas [64,65]. The underlying neural mechanism may lie in the individual’s inability to integrate sensory data and their adapt behavior, accordingly fostering addiction [66]. Lastly, the posterior midcingulate cortex (PMCC), a part of the default mode network (DMN) that processes self-generated thoughts and sensorimotor control [45,67], has seen impairment in the context of digital addiction. This suggests that addictive symptoms might stem from impairments in the sensorimotor cortex, prompting compulsive behaviors akin to those exhibited in cocaine and alcohol addiction [68,69,70].

In conclusion, digital addiction in children and adolescents leads to structural brain changes, including reduced grey and white matter volume in various regions involved in executive function, reward processing, and sensorimotor activities, impacting cognitive capabilities and contributing to problematic smartphone use, internet gaming disorder, and internet addiction.

#### 4.2.2. Reduced Cortical Thickness

The present shows a reduced cortical thickness in the right lateral OFC and pars orbitalis, which is in line with previous findings [71,72,73]. A possible reason for the decrease is that the pars orbitalis and OFC regions play a key role in reward processing and decision making, and adolescents who have thinner cortical thickness of the para orbitalis and OFC regions may have higher impulsivity to short-term reward and poorer ability to inhibit themselves for a delayed reward, which leads to uncontrolled internet use [74,75].

#### 4.2.3. Altered Cerebral Blood Flow

In the present review, only one study reported an alteration in cerebral blood flow (CBF) in adolescents with IGD. However, there is a different pattern in the changed CBF in different brain areas, which are responsible for visual/auditory (temporal and occipital cortex), memory (parahippocampus, hippocampus, and amygdala), control (ACC), reward (PCC), and the executive system (prefrontal cortex) [76,77,78]. The increased/decreased CBF of these areas indicated the fact that as a behavioral addiction, IGD relates to a structural alteration in adolescents’ brains. Such findings also suggest that digital addiction shared a similar mechanism as substance addictions like cocaine and alcohol addiction [79].

### 4.3. The Most-Affected Brain Area—Prefrontal Lobe

**Figure 2:**
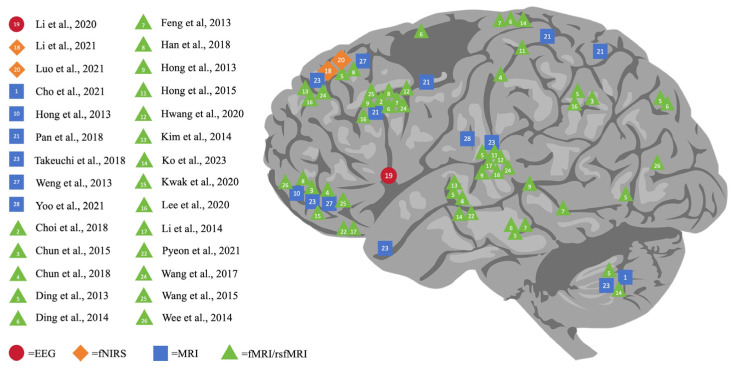
Neuroimaging finding of the impact of digital addiction on brain [5,6,7,20,21,22,23,24,28,29,30,31,32,33,34,35,36,37,38,39,40,41,42,43,44,45,46,47].

As shown in Figure 2, this comprehensive review outlines the varying impacts of DA on different brain regions, namely the cerebral cortex (including the prefrontal, parietal, temporal, and occipital lobes), subcortical structures (basal ganglia, thalamus, hippocampus amygdala, and nucleus accumbens), and the cerebellum. Verifying the existing review study [80], our review further indicated that DA resulted in significant alterations not only in the prefrontal zones but also in the temporoparietal, frontolimbic, and subcortical regions, specifically in adolescents with problematic gaming habits. Notably, it found that the prefrontal lobe is the area most affected by DA, making it a key focus of digital addiction studies. Concrete examples can be uncovered: 16 studies singled out specific prefrontal regions, namely the orbitofrontal cortex (OFC), supplementary motor area (SMA), and dorsolateral prefrontal cortex (DLPFC), in addition to the anterior cingulate gyrus. Such findings coincide with previous research.

A particularly poignant finding is that the subpar control processes in the prefrontal area which are identified in individuals with internet addiction (IA) might be associated with these people’s loss of control over their internet use [81]. This connection underscores the pre-eminent role that areas such as the DLPFC, OFC, and SMA play in cognitive control, emotional processing, and other high-level psychological functions [82,83]. Moreover, the intricate connection between the prefrontal cortex and the basal ganglia implies that damage to the prefrontal lobe could result in unregulated behavior in individuals struggling with digital addiction [84]. Thus, it is essential to consider the prefrontal lobe’s vulnerability to and pivotal role in DA.

In conclusion, this review underscores the fact that the prefrontal lobe is the most affected region in digital addiction, highlighting its consequential impact on cognitive control and emotional processing and underscoring its critical influence on resulting behaviors, thus warranting it as a key focus for future research and intervention strategies.

## 5. Conclusions and Limitations

Over the last decade, DA has become increasingly pervasive across all age brackets, and is accompanied by technological progress. This scoping review incorporates one study that includes samples from children aged between 3 and 8 years, as well as 27 other studies with samples of adolescents. In summary, two significant conclusions have been drawn from this comprehensive review. The first one being the detrimental effects of DA on the brains of children (ranging from birth to 18 years old). These effects are apparent in both their structural and functional aspects. The second finding involves the specific brain regions impacted, encompassing the brain’s cortex (frontal, parietal, temporal, and occipital lobes), cerebellum, and subcortical structures, such as the basal ganglia (containing the striatum and nucleus accumbens), the thalamus, and the hippocampus. It is worth noting that the prefrontal cortex is the most vulnerable region.

However, a few limitations are present in this scoping review. Firstly, the study’s sample size is quite limited, including only 28 studies. This limitation might be attributed to the novelty of the subject matter under consideration. Secondly, the constraints of time and multilingual sources have led to the sole inclusion of English articles in this review. Third, unlike systematic reviews, scoping reviews typically do not assess the quality of included studies. This means it is possible to include low-quality studies in the final review, which can impact the reliability and validity of its findings. Lastly, the direct impact of DA on the alterations in children’s brains remains unclear due to most of the included studies employing a cross-sectional design.

## 6. Implications for Practical Improvement and Future Studies

This scoping review provides several workable implications for combatting digital addiction among children. First, the findings highlight the essential role of parents in this effort. By reducing their own non-work related digital use and stimulating more face-to-face interaction with their children, parents could serve as exemplary models of a ‘digital citizen’. Secondly, it suggests educational institutions adopt a proactive stance. This includes communicating closely with parents to stay aware of children’s digital use, preventing digital addiction, and swiftly intervening with those children identified as high risk for digital addiction. This prompt action can substantially diminish the potential negative impacts of digital addiction on children’s brains. Lastly, this study also calls attention to the role of the government. To achieve the “early detection, early prevention and early intervention” target, the government needs to establish clear guidelines and policies concerning children’s digital use. These guidelines will shape the attitudes of teachers, parents, and children, thereby fostering children’s digital health and well-being. While a blanket ban on digital use in early childhood may appear comprehensive, it is vital to tailor these boundaries when considering various aspects of digital use, such as duration, devices, and content, as per previously established guidelines. This balanced approach should acknowledge the importance of digital tools in modern learning while addressing the potential risks.

The findings from this scoping review illuminate crucial areas for future studies. First, while current research has focused on comparing brain cognitive and structural differences relating to digital addictions, a scarcity of studies specifically examining the neural indices tied to digital addiction calls for more focused neurobiological research in future studies. Secondly, despite various mentioned effects, alterations in cerebral blood flow, potentially due to insufficient fNIRS studies, has only been reported in one instance. This suggests that future research would benefit from incorporating more fNIRS studies. This method would be useful in understanding the effects of digital addiction on children’s brains from a structural approach, thereby providing neuroimaging evidence from disparate viewpoints and complementing existing brain imaging techniques. Third, among the reviewed studies, only two concentrated on young children, leaving a significant research gap. Given the declining age of digital addiction onset, future studies should shift greater attention towards younger demographics, particularly young children. This refocused effort could enhance early detection and intervention strategies, possibly initiating in the early stages of childhood. Last but not least, the lack of longitudinal research examining the impact of digital addiction on children and teenagers must be addressed. To appreciate potential long-term neurodevelopmental effects of digital addiction, future studies are encouraged to adopt longitudinal designs. Prolonged tracking could foster an empirical basis for developing effective interventions to tackle digital addiction, particularly in youth. In sum, this review undeniably pushes for an expansion in the theoretical perspective regarding digital addiction, urging researchers to fill significant gaps in understanding its neural, structural, demographic, and longitudinal aspects.

## Figures and Tables

**Table 1:** The inclusion/exclusion criteria of this study.

Criteria	Inclusion	Exclusion
Scope of research	Empirical studies	Not empirical studies (reviews, theoretical studies, editorials) Secondary data analysis
Type of documents	Peer-reviewed scholarly journal articles	Not peer-reviewed scholarly journal articles
Language	English	Other languages than English
Participant	All participants: children aged from 0 to 18 years old Experiment group participants: diagnosed as at least one type of digital addiction (internet addiction, internet gaming addiction, cyber addiction, excessive internet use…) or self-reported to have suffered from typical components of at least one type of digital addiction	All participants: children aged over 18 years old Experiment group participants: children whose digital addiction or typical components of digital addiction were not mentioned Children with special needs
Method	Brain imaging technique (MRI, fNIRS, EEG, PET)	Only behavioral method
Period	January 2013–July 2023	Before 2013 and after July 2023
Research topic	How digital addiction influenced children’s brain function and brain structure	Articles that do not focus on children’s brain Articles that only presented behavior results

**Table 2:** Types of digital addiction in the 28 studies.

Types of Digital Addiction	Number
Internet gaming addiction	16
Internet addiction	6
Smartphone addiction	4
Heavy use of tablet	1
High media multitasking	1

**Table 3:** Types of neuroimaging technique of the 28 studies.

Types of Neuroimaging Technique	Number
EEG	1
MRI	6
fMRI	9
rsfMRI	10
fNIRS	2

## Data Availability

Not applicable.

## Appendix A

**Table A1:** Demographic information of the 28 studies.

No.	Author/Year	Country	Neuroimaging Technique	Participant’s Details					
				Sample Size	Age (Years)	Gender (%Male)	Addition Type	Diagnosis Criteria	Exclusion Criteria
1	Cho, I.H. et al., 2021 [28]	Republic of Korea	MRI	E: n = 20 C: n = 67	12–18 years old E: n = 16.2 ± 1.1 C: n = 15.3 ± 1.7	M: 56 F: 31	PSU	A total Smartphone Addiction Proneness Scale (SAPS) score of ≥42	(1) medical (i.e., diabetes); (2) neurological (i.e., convulsive disorder, head trauma); (3) psychiatric (i.e., affective disorder) disorders.
2	Choi, E.J. et al., 2018 [31]	Republic of Korea	fMRI	E: n = 12 C: n = 15	11–18 years old E: n = 13.83 ± 2.69 C: n = 15.33 ± 0.98	/	IGD	The Korean version of the Young Internet Addiction Scale (YIAS-K) score ≥50	(1) screened by the Kiddie-Schedule for Affective Disorders and Schizophrenia-Present and Lifetime Version (K-SADS-PL); (2) axis I psychiatric disorders including substance abuse, epilepsy or other neurological disorders, and past history of severe head trauma.
3	Chun, J.W. et al., 2015 [30]	Republic of Korea	fMRI	E: n = 16 C: n = 19	12–15 years old E: n = 13.63 ± 1.03 C: n = 13.37 ± 0.90	/	IGD	Estimated by the Korean Internet Addiction Proneness Scale (the K-scale)	(1) past or current major medical disorders (e.g., diabetes mellitus); (2) neurological disorders (e.g., seizure disorders, head injury); (3) psychiatric disorders (e.g., major mood disorders).
4	Chun, J.W. et al., 2018 [38]	Republic of Korea	fMRI	E: n = 38 C: n = 38	12–18 years old E: n = 14.95 ± 1.45 C: n = 1408 ± 1.28	M: 62 F: 14	SP	A total Smartphone Addiction Proneness Scale (SAPS) score of ≥42 or if the subscale scores for the disturbance of adaptive function, withdrawal, and tolerance ≥ 14, 12, and 13, respectively.	(1) past or current major medical disorders (e.g., diabetes mellitus); (2) neurological disorders (e.g., seizure disorders, head injury); (3) psychiatric disorders (e.g., major mood disorders).
5	Ding, W.N. et al., 2013 [40]	China	rsfMRI	E: n = 17 C: n = 24	14–17 years old E: 16.94 ± 2.73 C: 15.87 ± 2.69	M: 29 F: 12	IGA	Met the IGA criteria in the DSM-IV; assessed by the modified Young’s Diagnostic Questionnaire (YDQ)	(1) screened for psychiatric disorders with the Mini International Neuropsychiatric Interview for Children and Adolescents (MINI-KID); (2) a history of substance abuse or dependence, previous hospitalization for psychiatric disorders; (3) a history of major psychiatric disorders, such as schizophrenia, depression, anxiety disorder, and psychotic episodes.
6	Ding, W.N. et al., 2014 [35]	China	fMRI	E: n = 17 C: n = 17	E: 16.41 ± 3.20 C: 16.29 ± 2.95	M: 28 F: 6	IGA	Met the IGA criteria in the DSM-IV; assessed by the modified Young’s Diagnostic Questionnaire (YDQ)	(1) a history of head injuries or other major neurological disorders; (2) severe medical or surgical illness; (3) diagnoses such as schizophrenia, major depression with psychotic features, bipolar disorder, or substance use disorder.
7	Feng, Q et al., 2013 [36]	China	fMRI	E: n = 15 C: n = 18	14–17 years old E: n = 16.94 ± 2.34 C: n = 16.33 ± 2.61	M: 27 F: 6	IGA	Met the IGA criteria in the DSM-IV; assessed by the modified Young’s Diagnostic Questionnaire (YDQ)	(1) screened for psychiatric disorders with the Mini International Neuropsychiatric Interview for Children and Adolescents (MINI-KID); (2) a history of substance abuse or dependence, previous hospitalization for psychiatric disorders; (3) a history of major psychiatric disorders, such as schizophrenia, depression, anxiety disorder, and psychotic episodes.
8	Han, X et al., 2018 [7]	China	rsfMRI	E: n = 26 C: n = 30	E: 16.81 ± 0.75 C: 17.00 ± 0.89	M: 56 F: 0	IGD	Met the IGD criteria the modified Young’s Diagnostic Questionnaire (YDQ)	(1) screened for psychiatric disorders with the Mini International Neuropsychiatric Interview for Children and Adolescents (MINI-KID); (2) a history of substance abuse or dependence, previous hospitalization for psychiatric disorders; (3) a history of major psychiatric disorders, such as schizophrenia, depression, anxiety disorder, and psychotic episodes.
9	Hong, S.B. et al., 2013 [33]	Republic of Korea	fMRI	E: n = 12 C: n = 11	E: 13.41 ± 2.31 C: 14.81 ± 0.87	M: 23 F: 0	IA(IGD)	Diagnosed by the Young Internet Addiction Scale (YIAS); self-report	screened by the Kiddie-Schedule for Affective Disorders and Schizophrenia-Present and Lifetime Version (K-SADS-PL).
10	Hong, S.B. et al., 2013 [22]	Republic of Korea	MRI	E: n = 15 C: n = 15	E: 13.33 ± 2.84 C: 15.40 ± 1.24	M: 30 F: 0	IA(IGD)	Diagnosed by the Young Internet Addiction Scale (YIAS); self-report	(1) screened by the Kiddie-Schedule for Affective Disorders and Schizophrenia-Present and Lifetime Version (K-SADS-PL); (2) axis I psychiatric disorders including substance abuse, epilepsy or other neurological disorders, and past history of severe head trauma.
11	Hong, S.B. et al., 2015 [34]	Republic of Korea	fMRI	E: n = 12 C: n = 11	E: 13.41 ± 2.31 C: 14.81 ± 0.87	M: 23 F: 0	IGD	Diagnosed by the Young Internet Addiction Test (YIAT); self-report	screened by the Kiddie-Schedule for Affective Disorders and Schizophrenia-Present and Lifetime Version (K-SADS-PL);
12	Hwang, H et al., 2020 [32]	Republic of Korea	fMRI	E: n = 42 C: n = 41	12–15 years old E: n = 14.6 ± 1.1 C: n = 14.8 ± 2.0	/	IGD	Assessed with the Structured Clinical Interview of the DSM-5 Clinician Version	(1) history of head trauma and psychiatric or medical diseases; (2) IQ < 70; (3) claustrophobia.
13	Kim, J.E. et al., 2014 [37]	Republic of Korea	fMRI	AIA = 15 NA = 15	E: 13.83 ± 0.83 C: 13.83 ± 0.83	/	IA	Categorized into “adolescent internet addicts(score > 50)” and “normal adolescents (score< 40)” by the Korean; Adolescent Internet Addiction Scale (K-AIAS) and the Internet Game Addiction Diagnostic Scale (IGADS)	(1) a score of short-form Wechsler Intelligence Scale for Children-III < 80; (2) major psychiatric disorders, such as schizophrenia, affective disorder, conduct disorder; (3) substance-related disorders by Kiddie-Schedule for Affective Disorders and Schizophrenia—Present and Lifetime Version—Korean Version.
14	Ko, M. et al., 2023 [43]	Republic of Korea	rsfMRI	E: n = 20 C: n = 27	13–18years old E: 14.80 ± 1.64 C: 15.96 ± 1.02	M: 19 F: 28	cyber addiction	Diagnosed with cyber addiction using the modified criteria for Internet gaming disorders in Section III of the DSM-5	(1) IQ < 70; (2) with a family history of psychosis or personality disorder; (3) with a psychiatric or neurological impairment and head trauma resulting in loss of consciousness (for the patients no major psychiatric disorders); (4) with history of smoking and alcohol intake; (5) without a history of psychotropic prescription.
15	Kwak, K.H. et al., 2020 [41]	Republic of Korea	rsfMRI	E: n = 14 C: n = 12	E: n = 17.1 ± 0.3 C: n = 16.5 ± 1.2	/	IGD	Met the IGD criteria in the DSM-V	had not received any treatment or interventions, such as cognitive behavior therapy or psychiatric medications
16	Lee, J. et al., 2020 [42]	Republic of Korea	rsfMRI	E: n = 17 C: n = 18	12–15 years old E: n = 13.7 ± 0.9 C: n = 13.4 ± 1.0	M: 35 F: 0	IGD	Assessed through a structured interview, including the Korean Internet Addiction Proneness scale	(1) if their main purpose of using Internet were other than playing online game such as social networking and watching videos; (2) had history of current or past psychiatric disorders, neurological illness, traumatic brain injury, any radiological contraindications for MRI scanning.
17	Li, B. et al., 2014 [21]	China	fMRI	E: n = 18 C: n = 23	E: 15.1 ± 1.4 C: 15.2 ± 0.5	/	IA	Met the IA criteria the modified Young’s Diagnostic Questionnaire (YDQ); interviewed by two psychiatrists according to DSM-IV.	fulfilled any DSM-IV axis I disorders
18	Li, H. et al., 2021 [5]	China	fNIRS	heavy user: n = 16 non-user: n = 8	4–6.3 years old non-user: 5.03 ± 0.41 heavy user: 4.80 ± 0.68	M: 21 F: 17	heavy tablet use	Divided into three groups (i.e., heavy-user, low-user, and non-user) by Home Learning Environment and Practice Survey (HLEP)	low-user group
19	Li, Q. et al., 2020 [20]	China	EEG	E: n = 32 C: n = 32	E: 15.81 ± 1.68 C: 15.91 ± 1.73	M: 57 F: 6	IGD	Met the IGD criteria in the DSM-V; met the IGD criteria in the YDQ	(a) BDI scores > 13; (b) BAI scores > 15; (c) Axis I psychiatric disorders; (d) head injury or history of trauma;
20	Luo, J. et al., 2021 [47]	China	fNIRS	HMM: n = 12 LMM: n = 10	14–17 years old HMM: n = 16.17 ± 0.58 LMM: n = 15.90 ± 1.10	M: 7 F: 15	media multitasking	Snowball sampling via peers; divided into two groups (i.e., HMMs and LMMs) based on their scores on the media multitasking scale (MMS) (>12 into HMMs, <10 into LMMs)	/
21	Pan, N. et al., 2018 [23]	China	MRI	67	15.54 ± 0.14	M: 67	IGD	Assessed the severity of the tendency to IGD by internet addiction task (IAT); self-reported that spending more than 80% online time on game	(1) alcohol abuse or drug dependence; (2) existence of any neurological psychiatric disease such as insomnia, migraines, tinnitus, and attention deficit hyperactive disorder; (3) history of physical illness such as brain trauma, brain tumor, or epilepsy assessed according to clinical evaluations and medical records; (4) MRI contradiction; and visible abnormalities on conventional MRI.
22	Pyeon, A. et al., 2021 [39]	Republic of Korea	rsfMRI	E: n = 47 C: n = 46	13–18 years old E: n = 16.2 ± 1.1 C: n = 15.3 ± 1.7	M: 51 F: 42	PSU	A total Smartphone Addiction Proneness Scale (SAPS) score of ≥42 or if the subscale scores for the disturbance of adaptive function, withdrawal, and tolerance ≥ 14, 12, and 13, respectively.	adolescents with major medical disorders, neurological, or psychiatric disorders
23	Takeuchi, H. et al., 2018 [29]	Japan	MRI	Cross-sectional imaging analyses: 284 longitudinal imaging analyses: 223	Cross-sectional imaging analyses: 11.2 ± 3.1 longitudinal imaging analyses: 14.2 ± 3.0		Excessive internet use	categorized by self-report of current internet use habits	/
24	Wang, L. et al., 2017 [44]	China	rsfMRI	E: n = 26 C: n = 43	E: 15.0 ± 1.3 C: 15.1 ± 0.5	/	IA	Met the IA criteria the modified Young’s Diagnostic Questionnaire (YDQ); interviewed by two psychiatrists according to DSM-IV.	(1) fulfilled any DSM-IV axis I disorders; (2) alcohol, nicotine or any other substance abuse.
25	Wang, Y. et al., 2015 [45]	China	rsfMRI	E: n = 17 C: n = 24	14–17 years old E: n = 16.94 ± 2.73 C: n = 15.87 ± 2.69	/	IGD	Met the IGD criteria in the DSM-IV according to the modified Young’s Diagnostic Questionnaire (YDQ)	(1) a history of substance abuse or dependence; (2) previous hospitalization for psychiatric disorders; (3) history of major psychiatric disorders, such as schizophrenia, depression, anxiety disorder, and psychotic episodes.
26	Wee, C.Y. et al., 2014 [46]	America	rsfMRI	E: n = 17 C: n = 16	E: 17.3 ± 2.6 C: 17.7 ± 2.5	M: 29 F: 4	IAD	Assessed by the modified Young’s Diagnostic Questionnaire (YDQ); self-report and parents’ interview	(1) a history of comorbid psychiatric and non-psychiatric disorders, such as anxiety disorder, depression, compulsivity, schizophrenia, autism, or bipolar disorder; (2) a history of substance abuse or dependency; (3) a history of physical disorders related to the motion, digestive, nervous, respiratory, circulation, endocrine, urinary, and reproductive systems; (4) pregnancy or menstrual period in women during the day of scanning
27	Weng, C. et al., 2013 [6]	China	MRI (VBM TBSS)	E: n = 17 C: n = 17	E: 16.25 ± 3.02 C: 15.54 ± 3.19	M: 6 F: 28	OGA	Met the IA criteria the modified Young’s Diagnostic Questionnaire (YDQ);	(1) subjects with substance (alcohol, nicotine, or drug) abuse or dependence; (2) existence of neurologic or medical disorders (brain tumor, epilepsy, etc.); (3) a history or current episode of major psychiatric disorders, such as depression, anxiety disorder, schizophrenia, or psychotic episodes.
28	Yoo, J.H. et al., 2021 [24]	Republic of Korea	MRI	E: n = 20 C: n = 68	12–18 years old E: n = 16.2 ± 1.1 C: n = 15.3 ± 1.7	M: 57 F: 31	PSU	A total Smartphone Addiction Proneness Scale (SAPS) score of ≥42	(1) IQ < 70; (2) currently suffering from any major medical disorders (e.g., diabetes mellitus); (3) neurological disorders (e.g., seizure disorders, head injury); (4) psychiatric disorders (e.g., major depressive disorder, anxiety disorders, and ADHD).

IGD = internet addiction disorder; IA = internet addiction; IAD = internet addiction disorder; OGA = online gaming addiction; SP = excessive smartphone use; PSU = problematic smartphone use; EEG = Electroencephalogram; MRI = Magnetic Resonance Imaging; fMRI = functional Magnetic Resonance Imaging; rsfMRI = resting-state functional Magnetic Resonance Imaging; fNIRS = functional Near-Infrared Spectroscopy.

## Appendix B

**Table A2:** Research details of the 28 studies.

No.	Author/Year	Neuroimaging Technique	Addiction Type	Research Design		Research Result			Limitation
				Research Paradigm (If Had)	Behavioral Assessment (Questionnaire)	Type of Influence	Major Brain Area (Alteration)	Specific Outcomes	
1	Cho, I.H. et al., 2021 [28]	MRI	PSU	/	Beck Depression Inventory (BDI); The Brief Self-Control Scale (BSCS); the Korean-Weschsler Adults Intelligence Scale (K-WISC-IV).	structural	Cerebellar (↓volume)	A significantly smaller volume of the superior cerebellar peduncle (SCP) was found in the PSU group. The volume of the SCP were negatively correlated with the SAPS score.	need follow-up studies with two groups matching for these demographic variables; a further assessment of the changes in the volume or function of other brain regions; limited by the cross-sectional study design; no PSU subgroups
2	Choi, E.J. et al., 2018 [31]	fMRI	IGD	button reaction: two buttons to estimate whether the short phrase matched the characteristics of self/Admiral Yi/participant’s game character	IQ: Korean Educational Development Institute’s Korean Wechsler Adult Intelligence Scale(K-WAIS) (over 16) Korean Educational Development Institute’s Wechsler Intelligence Scale for Children-Revised (KEDI-WISC) (under 16); KIMS frontal executive function test; self-reported questionnaires for the recent use of internet game; Children’s Depression Inventory; Oiettolie Propensity Scale	functional	BA10, BA 32: prefrontal (↑activation under specific conditions)	A significant activation was found in medial prefrontal (MPFC) and ACC in IGA group when thinking about their own game characters than themselves. The ACC activation was correlated with the severity of IGA.	small sample size; may be influenced by the type of games
3	Chun, J.W. et al., 2015 [30]	fMRI	IGD	button reaction: three button that categorized neutral words, negative emotion words and neutral words	the Conners–Wells’ Adolescent Self-Report Scale-Short Version (CASS-S); the Korean-Wechsler Intelligence Scale for Children, 4th edition (K-WISC IV); Beck Depression Inventory (BDI);	functional	BA11, BA31: Prefrontal (↓activation)	The IGD group showed a reduced activation in the right OFC and in the dorsal anterior cingulate cortex (dACC) during the swear word condition. IGD were negatively correlated with activity in the right amygdala toward swear words.	not control the effect of word frequency on behavioral and neural responses; not consider the positive aspect of swearing related to the tolerance of pain, group solidarity and funny words; unable to analyze objective variables related to Internet gaming; not consider the various psychological and environmental variables of participants
4	Chun, J.W. et al., 2018 [38]	rsfMRI	excessive smartphone use (SP)	/	the Korean Internet Addiction Proneness Scale (the K-scale); the Beck’s Depression Inventory (BDI); the Beck’s Anxiety Inventory (BAI); the Korean-Wechsler Intelligence Scale for Children, 4th edition (K-WISC- IV)	functional	BA9: Prefrontal (↓FC)	Compared with HC, lower functional connectivity between the right OFC and NAcc, and between the left OFC and MCC, were found in the SP.	not consider various psychological and environmental variables of participants; not consider the main contents of the smartphone use
5	Ding, W.N. et al., 2013 [40]	rsfMRI	IGA	/	the Chen Internet Addiction Scale (CIAS) the Self-Rating Anxiety Scale (SAS) the Self-rating Depression Scale (SDS) the Barratt Impulsiveness Scale-11 (BIS-11)	functional	temporal, parietal, prefrontal (↑↓FC)	Increased FC in the bilateral cerebellum posterior lobe and middle temporal gyrus, and reduced FC in the bilateral inferior parietal lobule and right inferior temporal gyrus were found in IGA adolescents. Connectivity with the PCC was positively correlated with CIAS scores in the right precuneus, posterior cingulate gyrus, thalamus, caudate, NAcc, SMA, and lingual gyrus. It was negatively correlated with the right cerebellum anterior lobe and left superior parietal lobule.	use of self-report measures; small sample size; limited by the cross-sectional study design
6	Ding, W.N. et al., 2014 [35]	fMRI	IGA	Go/no-go task;	the Chen Internet Addiction Scale (CIAS) the Self-Rating Anxiety Scale (SAS) the Self-rating Depression Scale (SDS) the Barratt Impulsiveness Scale-11 (BIS-11)	functional	prefrontal, temporal, parietal (Abnormal hyperactive neural responses)	Compared to the control group, neural responses in the left superior MFG, right superior/middle frontal gyrus, right ACC, left inferior parietal lobule, left precentral gyrus, left precuneus and cuneus, the bilateral middle/inferior temporal gyrus, and right superior parietal lobule were more hyperactive during No-Go trials. Abnormal hyperactive function of the left superior medial frontal gyrus was positively associated with BIS and CIAS total score across IGA participants.	short task; no behavioral group difference but can be detected by fMRI; brain function may be influenced by social interaction; unclear direct causal relationship between Internet addiction and the impaired executive control ability
7	Feng, Q et al., 2013 [36]	fMRI	IGD	/	the Chen Internet Addiction Scale (CIAS) the Self-Rating Anxiety Scale (SAS) the Self-rating Depression Scale (SDS) the Barratt Impulsiveness Scale-11 (BIS-11)	structural	prefrontal, temporal, parietal (↓↑CBF)	Increased CBF in the left inferior temporal lobe, left para hippocampal gyrus and amygdala, right medial frontal lobe/ACC bilateral insula, right middle temporal gyrus, right precentral gyrus, left SMA, left cingulate gyrus, and right inferior parietal lobe. Decreased CBF was found in the left middle temporal gyrus, left middle occipital gyrus, and right cingulate gyrus were found in IGD groups.	limited sensitivity of ASL-MRI; limited by the cross-sectional study design; small sample size
8	Han, X et al., 2018 [7]	rsfMRI	IGD	/	the Chen Internet Addiction Scale (CIAS) the Self-Rating Anxiety Scale (SAS) the Self-rating Depression Scale (SDS) the Barratt Impulsiveness Scale-11 (BIS-11)	functional	prefrontal; parietal (↓FC)	The resting-state FC between the left medial OFC and the putamen was significantly lower in IGD group. The impairment in the prefrontal–striatal circuits may have an impact on impulsive behavior of IGD subjects. The FC values of IGD group were negatively associated with the BIS-11 scores. Functional alternations were observed in several prefrontal regions (including the right medial OFC, the bilateral SMA and the left ACC) and basal ganglia regions (the bilateral putamen) in addictive disorders, including IGD.	small sample size; only male participants; lack correct for multiple comparisons to control the false-positive error
9	Hong, S.B. et al., 2013 [33]	fMRI	IA(IGD)	/	IQ test	functional	prefrontal; parietal (↓FC)	IGD was related to reduced functional connectivity in cortico–striatal circuits; Impaired connection in cortico–subcortical circuits (24%with prefrontal and 27% with parietal cortex of IA adolescents were found.	small sample size; unclear validity of the IA measurement; may be influenced by subthreshold-level symptoms of comorbid mental conditions; collection of a broader range of clinical information; limited by the cross-sectional study design
10	Hong, S.B. et al., 2013 [22]	MRI	IA(IGD)	/	IQ test	structural	BA11: prefrontal (↓cortical thickness)	IA adolescents exhibited a reduced thickness in the right lateral OFC. Pars orbitalis, which is laterally adjacent to the lateral OFC, showed significant cortical thinning in adolescents with internet addiction.	a different age distribution between the groups; lack measurement of the duration of internet addiction; limit generalizability to other subtypes of internet addiction
11	Hong, S.B. et al., 2015 [34]	fMRI	IA(IGD)	/	IQ test	functional	parietal (↓FC)	Adolescents with IGD showed significantly reduced dorsal putamen FC with the posterior insula-parietal operculum. Functional connectivity between the dorsal putamen and bilateral primary somatosensory cortices in adolescents with IGD showed a positive correlation with internet game time.	small sample size; less severe symptoms of addiction; unclear validity of the IGD measurement (YIAT)
12	Hwang, H et al., 2020 [32]	fMRI	IGD	/	the Korean Wechsler Intelligence Scale for Children (K-WISC); Korean version of DuPaul’s ADHD Rating Scale (K-ARS); Young Internet Addiction Scale (YIAS); Children’s Depression Inventory (CDI); the Beck Anxiety Inventory (BAI); Family relationships: the relationship domain of the Family Environmental Scale (FES-R)	functional	prefrontal (↓FC)	The FC from the left cingulate to both lentiform nuclei was decreased and negatively correlated with the YIAS scores.	small sample size; the IGD measurement was not specifically for IGD; limited by the cross-sectional study design
13	Kim, J.E. et al., 2014 [37]	fMRI	IA	a right–left discrimination task in which each task belongs to performance feedback (PF), social reward (SR) (such as compliments), monetary reward (MR), and no reward (NR), respectively	/	functional	prefrontal, temporal (↓↑activation)	Almost no activation was found in AIA’s subcortical system, self-related brain region, and other brain areas for the three contrasts. AIA showed significant activation in the dorsolateral prefrontal cortex for the PF-NR contrast and the negative correlation was found between the level of activation in the left superior temporal gyrus (BA 22) and the duration of Internet game use per day in AIA.	not exclude the influence of personality or temperament; old type of SPM2 software; did not show OFC activation
14	Ko, M. et al., 2023 [43]	rsfMRI	cyber addiction	/	Internet Addiction Test (IAT) Korean Internet Addiction Scale (K-scale) Korean Internet Game Addiction Scale (G-scale) Korean Smartphone Addiction Scale (S-scale)	functional	temporal, parietal, hippocampus (↑FC)	Increased positive rsFC between the left insular−right middle temporal gyrus, the right hippocampus−right precentral gyrus, the right amygdala−right precentral gyrus, and right parietal operculum cortex. Increased negative rsFC between the left NAcc−right cerebellum crus II and right cerebellum VI.	overlooked other possible resting-state networks; limited by the cross-sectional study design; lack investigation of quantitative factors such as Internet use time and addiction period; insufficient evaluation and consideration of the symptoms of ADHD
15	Kwak, K.H. et al., 2020 [41]	rsfMRI	IGD	/	the Young Internet Addiction Scale (YIAS); Child Behavior Checklist (CBCL); Child Depressive Inventory (CDI); Beck Anxiety Inventory (BAI); Korean ADHD Rating Scale (K-ARS)	functional	prefrontal (hyperactivity)	Compared to pro-gamers, IGD adolescents showed hyperactivity within the left OFC.	small sample size; few assessment tools used to assess potential environmental factors; more objective criteria to estimate IGD; not consider different motivations, self-efficacy, and self-worth at the beginning
16	Lee, J. et al., 2020 [42]	rsfMRI	IGD	/	Barratt Impulsiveness Scale version 11(BIS-11); Conners-Wells Adolescent Self-Report Scale; Beck Depression Inventory (BDI); Beck Anxiety Inventory (BAI).	functional	frontal, temporal, parietal (FC)	Aberrant FC of salience network (SN) and default mode network (DMN) with the left posterior superior temporal sulcus (pSTS) were found in adolescents with IGD. FC between SN and pSTS correlated with proneness to Internet addiction and self-reported cognitive problems.	limited by the cross-sectional study design; the effects of depression still cannot be completely excluded; small sample size; use of self-report measures to evaluate executive function
17	Li, B. et al., 2014 [21]	fMRI	IA	Go-Stop task	/	functional	BA47: frontal (↓activation)	The accuracy of IA group was significantly lower than the control group during the 50 ms, 150 ms and 250 ms delay task. The activation of the corn brain regions in the response inhibition network (including the IFG and the striatum) in the IA group was weaker than the control group. The response inhibition was impaired in IA adolescents.	/
18	Li, H. et al., 2021 [5]	fNIRS	heavy tablet use	Dimensional Change Card Sort Task (DCCS)	/	structural	BA9: Prefrontal	The accuracy of non-users was significantly higher than heavy users in DCCS task; Before the 7th second, a significant increase in HbO and decrease in HbR were found in BA9 of non-users. During 12th second to 20th second, a significant decrease in BA9 were found in heavy users during DCCS task.	small sample size; fNIRS data disturbed by movement during the DCCS task; unbalanced allocation of boys and girls in different groups; no indication of any permanent damage in BA 9 for those heavy users
19	Li, Q. et al., 2020 [20]	EEG	IGD	Go/no-go task; gambling task;	SSS-V; BIS-11; BIS/BAS;	functional	prefrontal	IGD adolescents had a higher degree of sensation seeking and novelty seeking and were more likely to lead to negative outcomes. High IGD symptoms were related to low accuracy and reduced P3 amplitude in the no-go task. The IGD group demonstrated lower feedback-related negativity (FRN) magnitude after positive outcomes than the control group.	classification and diagnosis of IGD need more caution; dynamic regulation of the two systems were not examined due to the research design
20	Luo, J. et al., 2021 [47]	fNIRS	media multitasking	2-back; color Stroop; number-letter;	the Behavior Rating Inventory of Executive Function (Second Edition, BRIEF 2); Dysexecutive Questionnaire (DEX);	functional	BA9: Prefrontal (↑activation)	Executive function (working memory, inhibitory control, dysexecutive) of the HMMs was more impaired than the LMMs through the self-reported measurements. Prefrontal activation of the HMMs was greater than the LMMs during 2-back and Color Stroop task, which indicated that the HMMs’ reduced effectiveness in the brain areas responsible for executive function.	small sample size; lack counterbalance; limit task quantity for each executive function domain; no significant differences between HMMs and LMMs in the self-reported measure of shifting; consider avoiding the practice effects; did not consider the full continuum of media multitaskers
21	Pan, N. et al., 2018 [23]	MRI	IGD	/	Internet addiction test (IAT); Standard Raven’s Progressive Matrices; the self-rating anxiety scale (SAS); the self-rating depression scale (SDS)	structural	frontal, parietal (↓volume)	IGD tendency showed a negative correlation with the gray matter volumes (GMVs) of the bilateral postCG, the left preCG, the left posterior midcingulate cortex (pMCC), and the right MFG. High IAT score subgroup(score > 50) had lower GMVs of the bilateral postCG, the left preCG, the left pMCC, and the right MFG than low IAT score subgroup (score ≤ 50). Cognitive control was related to the IGD tendency.	unclear causality of the correlation between the brain GMV and IAT; lack objective method to assess IGD tendency and accurate measure of the total time of online games; only male participants
22	Pyeon, A. et al., 2021 [39]	rsfMRI	PSU	/	the Korean-Weschsler Adults Intelligence Scale (K-WISC-IV); self-control: the Brief Self-Control Scale (BSCS)	functional	frontal, limbic areas (↓rsFC)	The PSU group showed a reduced rsFC between the right inferior frontal gyrus and limbic areas, including the bilateral para-hippocampal gyrus, the left amygdala, and the right hippocampus. The rsFC of in frontolimbic was negatively associated with the severity of PSU and the degree of self-control.	not consider the primary reasons for smartphone use; no PSU subgroups; limited by the cross-sectional study design
23	Takeuchi, H. et al., 2018 [29]	MRI	Excessive internet use	/	IQ: the Japanese version of the Wechsler Adult Intelligence Scale-Third Edition (WAIS_x0002_III) (over 16) the Wechsler Intelligence Scale for Children-Third Edition (WISC-III) (under 16);	structural	prefrontal, temporal, cerebellum (↓volume)	Excessive internet use was correlated with decreased rGMV in certain areas (orbitofrontal and lateral prefrontal areas, the insula, the putamen, the pallidum, the thalamus, medial temporal areas, the anterior temporal pole, and the cerebellum) which was related to attention, executive function, language processing, emotion, and reward.	unclear direct causal relationship between the decreased rGMV and frequent internet use; not exclude the influence of certain individual tendencies toward internet use; not gather information regarding the type of internet use
24	Wang, L. et al., 2017 [44]	rsfMRI	IA	/	/	functional	frontal, parietal (↓FC)	Reduced inter-hemispheric functional connectivity of the right fronto–parietal network (FPN), whereas increased intra-hemispheric functional connectivity of the left FPN; Reduced functional connectivity in the dorsal medial prefrontal cortex (mPFC) of the anterior default mode network (DMN); Reduced functional connectivity between the salience network (SN) and DMN.	short duration of rs-fMRI scan; not directly explore the brain-behavior relationships; specific to adolescents with IA
25	Wang, Y. et al., 2015 [45]	rsfMRI	IGD	/	the Chen Internet Addiction Scale (CIAS) the Self-Rating Anxiety Scale (SAS) the Self-rating Depression Scale (SDS) the Barratt Impulsiveness Scale-11 (BIS-11)	functional	frontal (↓FC)	Compared to the control group, the IGD group exhibited decreased voxel-mirrored homotopic connectivity (VMHC) between the left and right superior frontal gyrus (orbital part), IFG (orbital part), MFG and superior frontal gyrus.	small sample size; the effects of methodological symmetry cannot be completely eliminated; no direct comparisons were made with other IA subgroups
26	Wee, C.Y. et al., 2014 [46]	rsfMRI	IAD	/	Young Internet Addiction Scale (YIAS); Barratt Impulsiveness Scale-11 (BIS-11) Time Management Disposition Scale (TMDS) Strengths and Difficulties Questionnaire (SDQ) McMaster Family Assessment Device (FAD)	functional	frontal, occipital, and parietal (↓FC)	Compared to the HC group, IAD group exhibited the disrupted FC between regions located in the frontal (right middle OFC), occipital (the left fusiform gyrus), and parietal (the left angular gyrus) lobes. The affected connections are long-range and inter-hemispheric connections. The observed regional abnormalities are correlated with the IAD severity and behavioral clinical assessments.	small sample size; only male participants; only self-reported assessment
27	Weng, C. et al., 2013 [6]	MRI (VBM TBSS)	OGA	/	The Young’s Internet Addiction Scale (YIAS); Barratt Impulsiveness Scale-11 (BIS-11)	structural	prefrontal (↓volume)	OGA exhibited that gray and white matter integrity were impaired in some areas of prefrontal lobe (the OFC, the SMA).	small sample size; limitations to VBM
28	Yoo, J.H. et al., 2021 [24]	MRI	PSU	/	The Korean Kiddie Schedule for Affective Disorders and Schizophrenia–Present and Lifetime version (K-SADS-PL); the Dickman Functional and Dysfunctional Impulsivity Inventory (DFII); the Patient Health Questionnaire-9 (PHQ-9); the Generalized Anxiety Disorder-7 (GAD-7);	structural	caudate (↓volume)	A decrease volume in both caudate were found in the PSU group. Left caudate volume was negatively correlated with impulsivity traits and SAPS scores.	significant differences in demographic variables such as age and gender; small sample size; not measured ADHD symptoms; lacked evidence from a task-based fMRI experiment

IGD = internet addiction disorder; IA = internet addiction; IAD = internet addiction disorder; OGA = online gaming addiction; SP = excessive smartphone use; PSU = problematic smartphone use; EEG = Electroencephalogram; MRI = Magnetic Resonance Imaging; fMRI = Functional Magnetic Resonance Imaging; rsfMRI = resting-state functional Magnetic Resonance Imaging; fNIRS = functional Near-Infrared Spectroscopy; OFC = orbitofrontal cortex; ACC = anterior cingulate cortex; MCC = midcingulate cortex; PCC = posterior cingulate cortex; NAcc = nucleus accumbens; SMA = supplementary motor area; IFG = inferior frontal gyrus; postCG = postcentral gyri; preCG = precentral gyri; MFG = middle frontal gyrus; FC = functional connectivity; CBD = cerebral blood flow; ↑ = increased; ↓ = decreased.
